# Treatment patterns and outcomes of second/third-line therapy in advanced non-small cell lung cancer with actionable genomic alterations (RECAP)

**DOI:** 10.1007/s10585-026-10417-x

**Published:** 2026-07-30

**Authors:** Hanxiao Chen, Xiangjiao Meng, Ling Cai, Wei Lei, Yu Tang, Xi Shi, Leilei Ma, Jun Zhao

**Affiliations:** 1 Department of Thoracic Oncology, Peking University Cancer Hospital and Institute, Beijing, China; 2 Department of Radiation Oncology, Shandong Cancer Hospital and Institute, Shandong First Medical University and Shandong Academy of Medical Sciences, Jinan, China; 3 Department of Radiation Oncology, Sun Yat-sen University Cancer Center, Guangzhou, China; 4 Department of Pulmonary and Critical Care Medicine, The First Affiliated Hospital of Soochow University, Suzhou, China; 5 Department of Thoracic Oncology, Liaoning Cancer Hospital and Institute, Shenyang, China; 6 Department of Oncology, The First Affiliated Hospital of Fujian Medical University, Fuzhou, China; 7 Department of Medical Affairs, Daiichi Sankyo (China) Holdings Co., Ltd, Shanghai, China

**Keywords:** Non-small-cell lung cancer, Epidermal growth factor receptor mutation, Real-world study, Second-line therapy, Targeted therapy

## Abstract

Treatment choices for metastatic non-small-cell lung cancer (NSCLC) after first-line targeted therapy progression are heterogeneous. This study aimed to characterize real-world treatment patterns and outcomes of advanced NSCLC harboring actionable genomic alterations (AGAs) in second- (2 L) or third-line (3 L) settings. This retrospective cohort study across six Chinese centers included stage IV NSCLC patients with confirmed AGAs. Therapies were divided into six categories: targeted monotherapy (T), targeted combinations (T+), chemotherapy monotherapy (C), chemotherapy combinations (C+), anti-angiogenic monotherapy (A), and other regimens (O). The primary outcome was the treatment pattern. Secondary outcomes included biomarker testing and effectiveness. A total of 658 patients were enrolled, with the majority harboring EGFR mutations (*n* = 590, 89.7%). In the 2 L-enrolled group (*n* = 602), T use declined from 75.3% in the 1 L setting to 49.3% in 2 L, while utilization of A + C increased to 16.5%. Within the EGFR-mutant subgroup, therapeutic sequences were highly dependent on 1 L TKI generation and T790M resistance status. Over half (51.1%) of T790M-negative patients continued T in 2 L following progression on 1 L first- or second-generation TKIs. Among the 3 L-enrolled patients (*n* = 56), A + C (35.7%) and C (14.3%) were predominant. The overall median real-world progression-free survival for the 2 L-enrolled group was 7.4 months in the 2 L setting (7.5 months for the EGFR-mutant subgroup) and 5.4 months in 3 L. This multicenter real‑world cohort predominantly comprised patients with EGFR‑mutant NSCLC, with smaller numbers of other AGA subtypes. Targeted therapies remain the cornerstone of later-line advanced NSCLC management. The prevalent real-world reliance on continued TKI therapy, even in T790M-negative patients, highlights the clinical dilemma of limited post-resistance options and the need to integrate emerging novel therapeutics.

**Supplementary Information:**

The online version contains supplementary material available at 10.1007/s10585-026-10417-x.

## Introduction

Lung cancer remains the leading cause of global cancer incidence and mortality, with the highest disease burden observed in China [1, 2]. Non-small-cell lung cancer (NSCLC) constitutes 80% to 85% of these cases, frequently presenting at advanced stages [3]. The identification of actionable genomic alterations (AGAs) has propelled a paradigm shift in NSCLC management, transitioning from histology-driven approaches to molecularly targeted therapies [4]. Notably, Chinese patients with NSCLC exhibit a higher prevalence of key driver mutations, such as epidermal growth factor receptor (EGFR) mutations (47.6% vs. 20.0%), compared to Western cohorts [5]. Consequently, AGA-directed tyrosine kinase inhibitors (TKIs) have been widely adopted as first-line therapy, substantially improving clinical outcomes [6, 7]. However, therapeutic efficacy is ultimately limited by acquired resistance. For instance, the EGFR T790M mutation mediates approximately 50% of resistance to first-generation EGFR-TKIs [8]. Next-generation TKIs address T790M-positive disease, but resistance emerges even with upfront use [9]. Furthermore, central nervous system (CNS) progression remains a formidable clinical challenge, with brain metastases in approximately 29% of metastatic NSCLC, especially in tumors harboring oncogenic drivers, leading to shorter progression-free survival (PFS) after TKI failure [10, 11].

For patients progressing after first-line targeted therapy, subsequent treatment is guided by molecular profile, prior response, and performance status [6]. The Chinese Society of Clinical Oncology (CSCO) and National Comprehensive Cancer Network (NCCN) guidelines recommend osimertinib for T790M-positive patients who have not previously received it [6, 7]; for T790M-negative or third-generation TKI-refractory patients, platinum-based chemotherapy, with or without bevacizumab, remains the standard [6, 7]. However, the evidence establishing these guidelines predominantly stems from rigorous randomized controlled trials (RCTs), which frequently exclude patients with poor performance status, severe comorbidities, or untreated brain metastases, thereby limiting their broad applicability in routine clinical practice [12]. In the real world, clinical outcomes with standard regimens are often modest. Anti-angiogenic therapy combined with chemotherapy yields a modest objective response rate (ORR) of 13.2% and a median PFS of 6.9 months in T790M-negative populations [13]. Emerging data suggest that immune checkpoint inhibitors (ICIs), particularly when combined with chemotherapy, may offer enhanced clinical benefit, achieving an ORR of 29.5% and a median PFS of 7.6 months [13]. Furthermore, the ORIENT-31 trial showed that adding anti-angiogenic agents to ICI-chemotherapy improved ORR from 25 to 45% and median PFS from 4.3 to 6.9 months in EGFR-TKI progressed patients [14].

Driven by these promising yet varied clinical trial signals, real-world treatment paradigms have rapidly diversified. Physicians frequently employ highly heterogeneous, and sometimes off-label, approaches, including TKI rechallenge, chemoimmunotherapy, and complex quadruplet regimens (chemotherapy, anti-angiogenics, and ICIs) [15–20], despite concerns regarding immune-related adverse events (irAEs). This heterogeneity is further exacerbated in China by pronounced disparities in healthcare infrastructure, diagnostic access, and drug affordability [15, 16]. Although previous real-world studies have documented these alternative treatment patterns [15–21], they present significant limitations. Most are constrained by small sample sizes, single-center designs [18], or rely on outdated cohorts that predate the widespread availability and national reimbursement of novel domestic ICIs and third-generation TKIs [15, 16]. More importantly, existing literature predominantly focuses on the immediate short-term efficacy of specific, isolated regimens (e.g., second-line ORR of a single combination) [17, 19, 22], often failing to capture the longitudinal treatment trajectory and the interplay between successive lines of therapy, particularly how front-line TKI selection and resistance-driven biomarker dynamics shape subsequent clinical pathways. Consequently, this leaves a critical knowledge gap regarding the survival outcomes and optimal sequencing strategies of a complex, multi-line treatment landscape.

This multicenter, retrospective study aimed to characterize treatment and biomarker testing patterns, and clinical outcomes in Chinese patients with advanced NSCLC receiving second- or later-line therapies. The analysis provides real-world insights into advanced NSCLC with AGAs to inform personalized treatment and guide clinical practice.

## Methods

### Study design and population

The RECAP study was a multicenter, retrospective, real-world study conducted at six centers across China. Patients diagnosed with advanced NSCLC who initiated second- or third-line systemic therapy between September 1, 2019, and December 31, 2022, were eligible. This enrollment window overlapped with important regulatory and reimbursement transitions for EGFR-TKIs in China, particularly the expanding approval and National Reimbursement Drug List (NRDL) coverage of third-generation agents.

Based on AGA testing, patients were classified into an AGA cohort (positive for EGFR, ALK, ROS proto-oncogene 1 [ROS1], eurotrophic tyrosine receptor kinase [NTRK], B-Raf proto-oncogene [BRAF], ret proto-oncogene [RET), or MET proto-oncogene, receptor tyrosine kinase [MET] exon 14 skipping alterations) or a non-AGA cohort. This analysis focused on treatment patterns and clinical outcomes in the 2 L/3L setting for the AGA cohort, particularly the EGFR-mutant subgroup.

Within the AGA cohort, patients were initially classified by therapy line at enrollment into the second-line (2 L-enrolled) or third-line (3 L-enrolled) groups, and further stratified by EGFR mutation status. Among the 2 L-enrolled patients with EGFR mutations, additional subgroups were defined based on prior TKI exposure and T790M status. The categories for this stratification were: (1) patients who received 1 L third-generation EGFR-TKIs; (2) patients who were treated with first- or second-generation EGFR-TKIs in the 1 L therapy and tested positive for the T790M mutation at the 2 L therapy; and (3) patients who initially received first- or second-generation EGFR-TKIs in the 1 L therapy but did not have the T790M mutation at the 2 L therapy. Furthermore, to capture a broader clinical perspective, the 2 L-enrolled group was also characterized by clinical factors, specifically the receipt of platinum-based chemotherapy and the presence of newly developed brain metastases in 2 L. Follow-up continued until October 30, 2023.

Eligible patients were required to meet the following criteria: age of 18 years or older at the time of diagnosis; histologically or cytologically confirmed stage IV NSCLC; presence of at least one AGA; and documented initiation of second- or third-line systemic therapy during the defined study period. Patients with confirmed EGFR, ALK, or ROS1 alterations must have received targeted therapy directed against these mutations in either the first- or second-line setting. Patients were excluded if they had previously participated in or were actively enrolled in a lung cancer clinical trial during the data collection window. Additional exclusion criteria included a concurrent diagnosis of another malignancy at the time of NSCLC diagnosis (except for non-metastatic non-melanoma skin cancers, carcinoma in situ, or benign neoplasms); development of a second primary malignancy within five years following the NSCLC diagnosis; histological diagnosis of small cell lung cancer, neuroendocrine carcinoma, or mixed small cell and non-small cell histology; or significantly incomplete medical records or other factors deemed by the investigators to preclude reliable analysis.

The study was conducted in compliance with the principles of the Declaration of Helsinki and adhered to the reporting standards outlined in the Strengthening the Reporting of Observational Studies in Epidemiology (STROBE) guidelines. Institutional review board approval was obtained at the lead study site (Peking University Cancer Hospital, approval number: 2024YW114). Given the retrospective design, the requirement for written informed consent was waived. The RECAP study is registered on ClinicalTrials.gov (NCT06617390).

### Data collection and variables

Patient-level data were retrospectively extracted from electronic medical records (EMR) and hospital information systems (HIS) across participating centers. The collected data included demographic characteristics (age, sex, smoking history), baseline disease features (TNM classification, clinical stage, histological subtype, pathological diagnosis, ECOG performance status, comorbidities), brain metastases status (newly developed), distant metastatic sites, prior treatments (definitive surgery, radiotherapy, first-line systemic therapy), and treatment-related details such as line of therapy, and treatment durations. Regimens were categorized into six groups: targeted monotherapy (T), targeted combination therapies (T+), chemotherapy monotherapy (C+), chemotherapy-based combinations without targeted agents (C+), anti-angiogenic monotherapy (A), and other regimens (O).

Biomarker testing patterns were also documented with respect to test timing, detection rates, methodologies such as next-generation sequencing (NGS) or polymerase chain reaction (PCR), and sample sources.

### Outcomes

Treatment patterns referred to the therapeutic regimens administered at enrollment and in subsequent treatment lines. The primary outcome of the RECAP study was the real-world distribution of different treatment options in NSCLC patients. The frequency and percentage will be presented to illustrate the proportion of different treatment regimens. Secondary outcomes included patterns of biomarker testing and clinical outcomes. The testing pattern for the biomarker included the timing, proportion, methods, and specimen types. Additionally, the positivity rate among the tested patients was calculated. Clinical outcomes included real-world effectiveness and safety profiles. Real-world effectiveness included real-world progression-free survival (rwPFS), time to treatment discontinuation (rwTTD), time to next treatment or death (rwTTNT), and real-world overall survival (rwOS). rwPFS was defined as the time from initiation of the current line of therapy to the earliest of: (1) first documented disease progression, (2) death from any cause, or (3) initiation of a subsequent line of systemic therapy in the absence of a recorded progression date. Disease progression was identified based on clinical documentation in electronic medical records, including radiology reports and physician notes, and reflects routine physician‑assessed progression in real‑world practice. rwTTD was defined as the time from therapy initiation to definitive treatment discontinuation or death, and rwTTNT as the time from therapy initiation to the start of the next systemic line or death. The detailed definitions of the effectiveness metrics are presented in the Supplementary Materials.

The safety profile included AEs, defined as any adverse medical event occurring in a participant while taking the medication, but not necessarily causally related to the treatment. All AEs were graded for severity according to the Common Terminology Criteria for Adverse Events (CTCAE). The collection of AEs was exclusively based on data documented in medical records during hospitalization and outpatient clinic visits.

### Statistical analysis

The full analysis set (FAS) comprised all eligible patients. The efficacy analysis set (EAS) comprised FAS patients with evaluable effectiveness data, and the safety set (SS) included FAS patients with ≥ 1 post-baseline safety assessment.

Categorical variables were summarized as frequencies and percentages. Continuous variables were described using the arithmetic mean, standard deviation (SD), median, interquartile range (IQR), and ranges. Missing outcomes were not imputed. Time-to-event endpoints were estimated using the Kaplan-Meier method, with 95% confidence intervals (CIs). Analyses were conducted using R version 4.2.1 (R Foundation, Vienna, Austria).

## Results

### Baseline characteristics

Between 2018 and 2022, 11,194 individuals with stage IV NSCLC were screened, of whom 4718 received 2 L or 3 L treatment between 2019 and 2022. A total of 658 patients with molecularly confirmed AGAs were ultimately included (Supplementary Fig. 1). While the eligible AGAs cohort comprised seven specific alterations (EGFR, ALK, ROS1, NTRK, BRAF, RET, and MET; Supplementary Table 1), the predominant proportion consisted of those with EGFR mutations (*n* = 590, 89.7%). Consequently, our analysis encompasses both the overall treatment landscape and the clinical outcomes of the entire AGA cohort, along with a detailed characterization of the EGFR-mutant subgroup.

Overall, the AGA cohort exhibited a mean age at enrollment of 59.3 ± 10.9 years, with a female predominance (57.9%). Adenocarcinoma was the primary histological subtype, accounting for 96.1% of cases. Intracranial metastases were present in 45.7% of patients (7.3% newly diagnosed), and most patients had additional distant metastases. In terms of treatment setting, the majority of patients were enrolled during 2 L therapy (*n* = 602, 91.5%), while the remainder initiated 3 L treatment. Detailed characteristics are shown in Table 1.

**Table 1:** Characteristics of patients with AGAs

Variable	All (*N* = 658)
Age (years), Mean ± SD	59.3 ± 10.9
Sex, *n* (%)	
Male	277 (42.1)
Female	381 (57.9)
Smoking history, *n* (%) [*n* = 624]	
Yes	166 (26.6)
No	458 (73.4)
ECOG performance status, *n* (%) [*n* = 339]	
0	88 (26.0)
1	195 (57.5)
2	39 (11.5)
3	11 (3.2)
4	6 (1.8)
Histological type, *n* (%) [*n* = 656]	
Squamous cell carcinoma	13 (2.0)
Adenocarcinoma	632 (96.3)
Adenosquamous carcinoma	8 (1.2)
Other	3 (0.5)
Presence of comorbidities, *n* (%)	432 (65.7)
History of intracranial metastases, *n* (%)	301 (45.7)
History of other distant metastatic sites, *n* (%)	643 (97.7)
Prior tumor surgery, *n* (%)	74 (11.3)
Prior tumor radiotherapy, *n* (%)	152 (23.1)
Newly developed brain metastases at enrollment, *n* (%)	48 (7.3)
EGFR mutation status, *n* (%)	
EGFR-mutant	590 (89.7)
Non-EGFR mutant	68 (10.3)

SD, standard deviation; ECOG, eastern cooperative oncology group; *EGFR*, epidermal growth factor receptor

### Treatment landscapes and longitudinal patterns in the AGA cohort

**Fig. 1:**
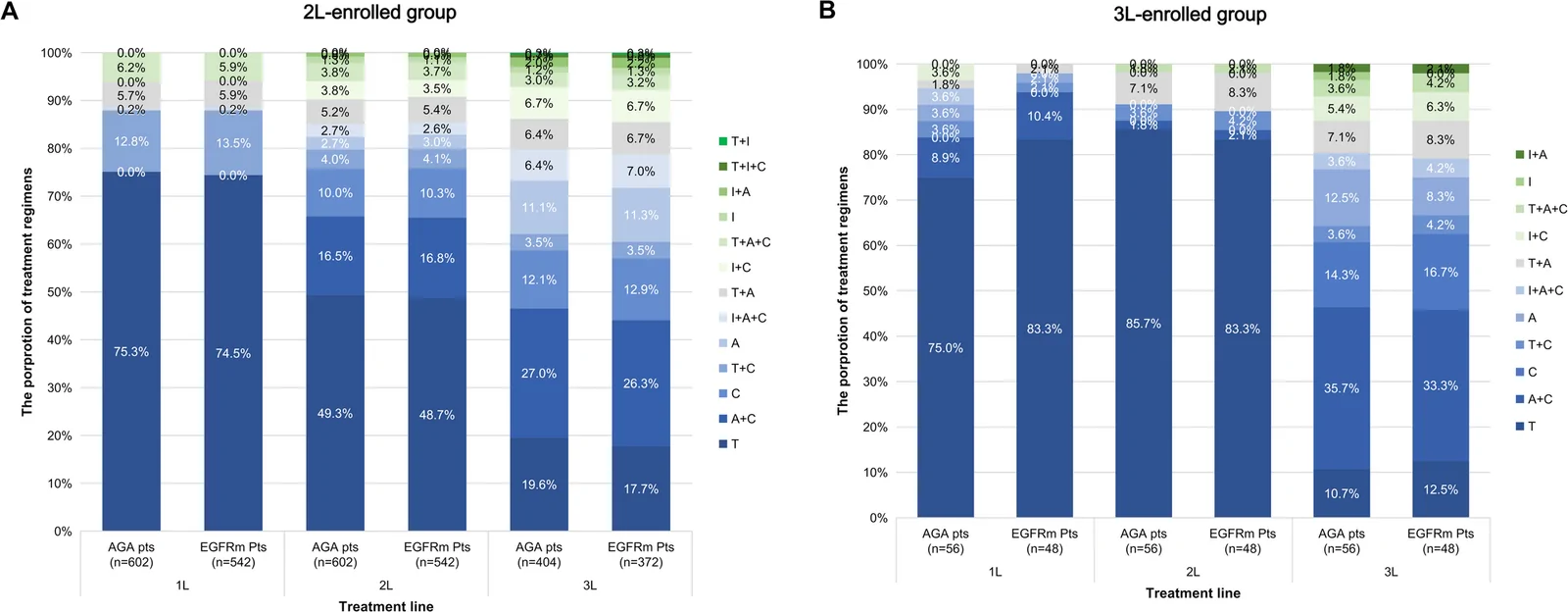
The real-world distribution of different treatment options for NSCLC patients.** A** 2 L-enrolled group **B** 3 L-enrolled group. 2 L, second line; 3 L, third line; AGA, actionable genomic alteration; EGFRm, EGFR mutations; T, targeted monotherapy; I, immunotherapy monotherapy; A, anti-angiogenic monotherapy; C, chemotherapy monotherapy

In the 1 L setting, T was the most common regimen among 2 L-enrolled patients (75.3%, 453/602), a pattern consistently observed in the EGFR-mutated subgroup (74.5%, 404/542) (Fig. 1A, Supplementary Tables 2 and 3). Upon transitioning to 2 L treatment, T use declined to 49.3% (297/602), whereas A + C (16.5%, 99/602) and C (10.0%, 60/602) became more prominent. A similar shift was observed among EGFR-mutated patients, with proportions of 48.7% (264/542), 16.8% (91/542), and 10.3% (56/542) (Fig. 1A, Supplementary Tables 2 and 3). Within this 2 L setting, further analysis of other subgroups revealed even more distinct therapeutic choices. Among patients receiving platinum-based chemotherapy (*n* = 194, 32.2%), A + C (41.2%, 80/194) and C (26.3%, 51/194) were the predominant choices, whereas T remained the mainstay (72.8%, 297/408) for those who did not receive platinum-based chemotherapy (*n* = 408, 67.8%) (Supplementary Table 4). Additionally, for patients with newly developed brain metastases (*n* = 46), T remained the most frequently utilized 2 L regimen (80.4%, 37/46) (Supplementary Table 5).

Returning to the longitudinal progression of the overall 2 L-enrolled population, the proportion of patients receiving T further decreased to 19.6% (79/404) as they advanced to the 3 L setting, while A + C increased to 27.0% (109/404). These trends remained consistent within the EGFR-mutated subgroup (T, 17.7% [66/372]; A + C, 26.3% [98/372]; Fig. 1A, Supplementary Tables 2 and 3).

Regarding the 3 L-enrolled group (*n* = 56), T was the predominant regimen during prior 2 L treatment (85.7%, 48/56), a pattern consistent with that observed in the EGFR-mutant subgroup (83.3%, 40/48). However, upon transitioning to the 3 L setting, T utilization markedly decreased to 10.7% (6/56) in the overall 3 L-enrolled group and 12.5% (6/48) in the EGFR-mutant subgroup. In contrast to this decline, the A + C regimen emerged as the most frequently administered 3 L therapy, accounting for 35.7% (20/56) of the overall 3 L-enrolled group and 33.3% (16/48) of the EGFR-mutant subgroup (Fig. 1B, Supplementary Tables 2 and 3).

### Treatment sequences within the EGFR-mutant subgroup

Within the 2 L-enrolled group (*n* = 542), the EGFR-mutant population was further analyzed according to the generation of 1 L EGFR-TKI and the resistance profile at progression. Among these patients, 136 received a third-generation TKI, while 404 received a first- or second-generation TKI in the 1 L setting.

**Fig. 2:**
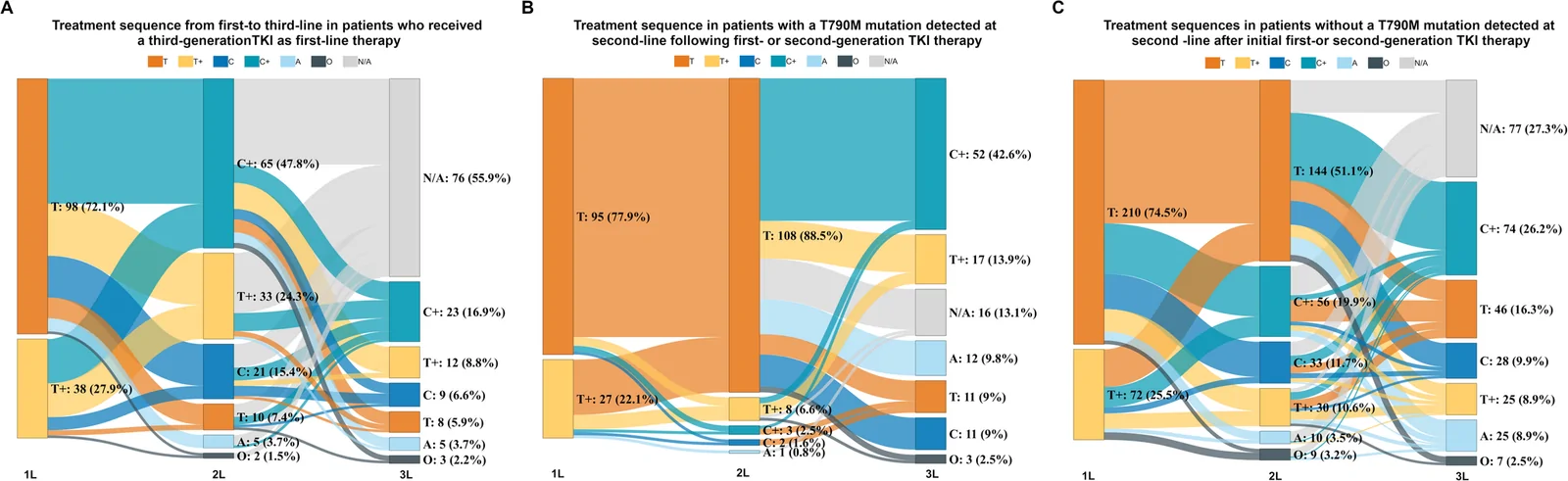
Sankey diagrams of treatment sequences in EGFRm 2 L-enrolled patients. **A** Treatment sequence from first- to third-line in patients who received a third-generation TKI as first-line therapy. **B** Treatment sequence in patients with a T790M mutation detected at second-line following first- or second-generation TKI therapy. **C** Treatment sequences in patients without a T790M mutation at second-line after initial first- or second-generation TKI therapy. 2 L, second line; EGFRm, EGFR mutations; TKI, tyrosine kinase inhibitor; N/A, no treatment regimen recorded

For patients initially treated with third-generation TKIs, the primary 2 L regimens were C+ (47.8%), T+ (24.3%), and C (15.4%). This distribution remained consistent in the subsequent post-progression setting, with C+ being the predominant regimen (Fig. 2A).

Among those who received 1 L first- or second-generation TKIs, therapeutic trajectories were further stratified by T790M mutation status. Of these, 122 patients were confirmed to harbor EGFR T790M mutations upon progression, with 88.5% subsequently receiving TKI monotherapy as their 2 L strategy. Over time, their treatments shifted primarily to C+ (42.6%), T+ (13.9%), and A (9.8%) (Fig. 2B). In contrast, for 282 patients without T790M mutations, over half (51.1%) continued on T therapy during 2 L treatment, while C+ (19.9%) and C (11.7%) served as alternative options. Following further progression, C + and T emerged as the most common regimens, accounting for 26.2% and 16.3%, respectively (Fig. 2C).

### rwPFS

**Fig. 3:**
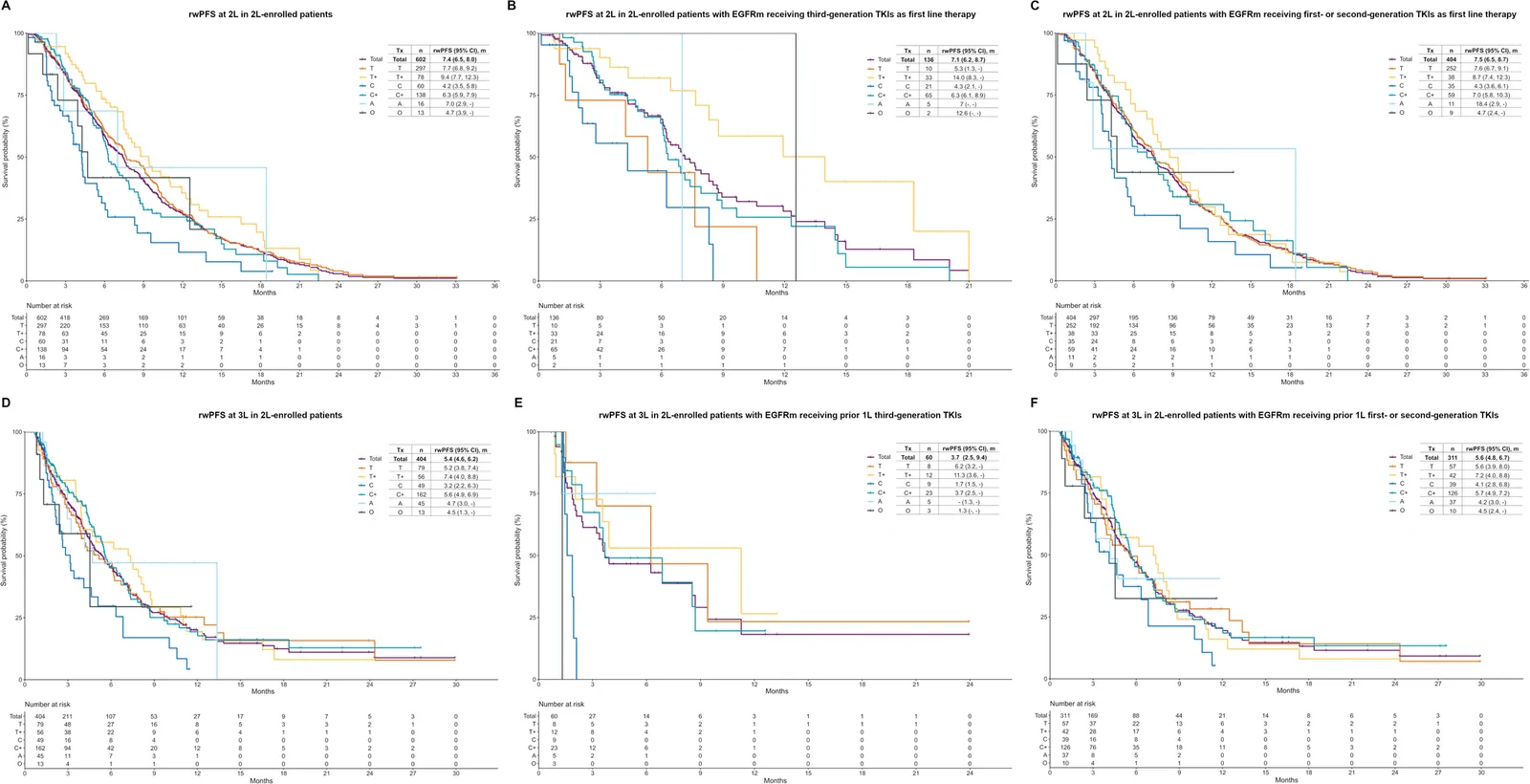
rwPFS in 2 L-enrolled patients. **A** rwPFS for 2 L-enrolled patients **B** rwPFS in EGFRm patients receiving third-generation TKIs as first-line therapy. **C** rwPFS for EGFRm patients receiving first- or second-generation TKIs as first-line therapy. **D** rwPFS at post-progression 3 L therapy. **E** rwPFS at 3 L in EGFRm patients receiving prior 1 L third-generation TKIs. **F** rwPFS at 3 L in EGFRm patients receiving prior 1 L first- or second-generation TKIs. rwPFS, real-world progression-free survival; 2 L, second line; EGFRm, EGFR mutations; 1 L, first line; 3 L, third line

With a median follow-up of 10.1 months (range, 0.03–38.2), the median rwPFS for the 2 L-enrolled group was 7.4 months (95% CI, 6.5–8.0) in 2 L treatment and 5.4 months (95% CI, 4.6–6.2) in 3 L treatment (Fig. 3 and Supplementary Table 6). Subgroup analyses of rwPFS, including patients with EGFR-mutant status, new brain metastases, and 2 L chemotherapy stratified by platinum exposure were shown in Fig. 3 and Supplementary Fig. 2. Within the EGFR-mutated subgroup, patients initially receiving third-generation TKIs followed by T + in 2 L treatment achieved a median rwPFS of 14.0 months (95% CI, 8.3-not estimable [NE]) (Fig. 3B). Notably, in a smaller subgroup of 11 EGFR-mutated patients initially treated with first- or second-generation TKIs followed by A in 2 L treatment, the median rwPFS was 18.4 months (95% CI, 2.9-NE) (Fig. 3C).

**Fig. 4:**
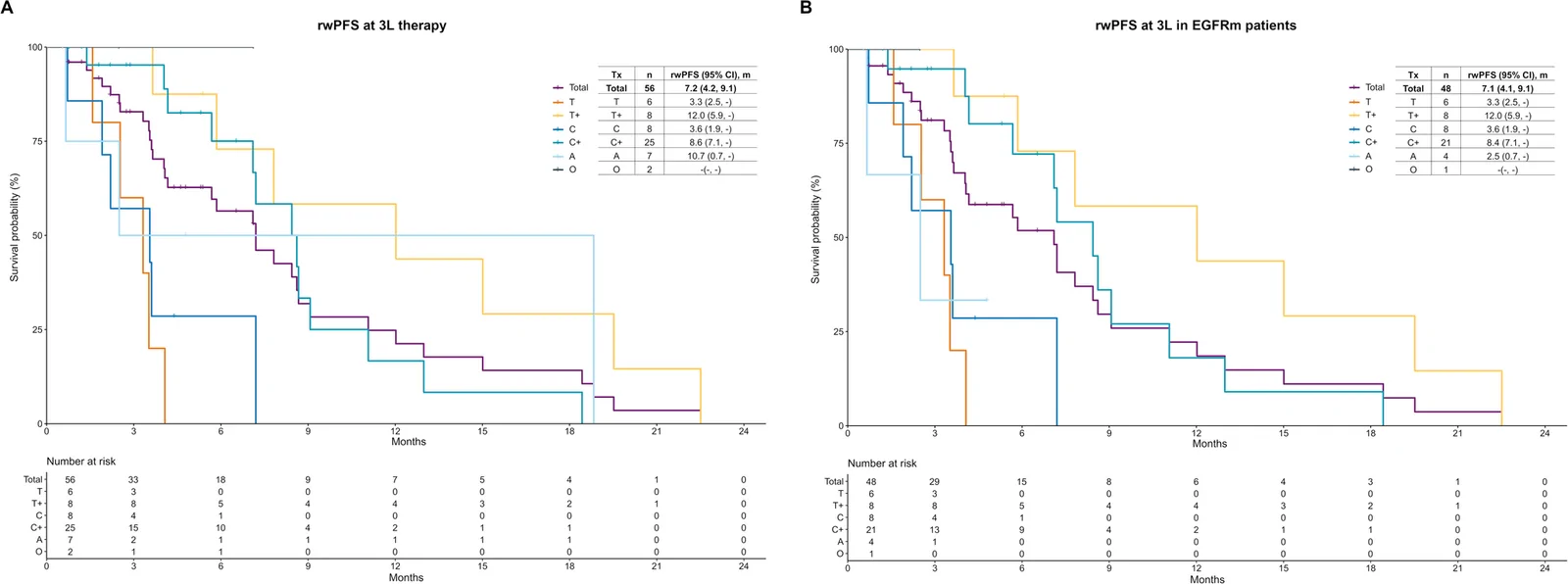
Real-world progression-free survival (rwPFS) in 3 L-enrolled patients. **A** rwPFS at 3 L therapy. **B** rwPFS at 3 L in EGFRm patients

The rwPFS outcomes for patients in the 3 L-enrolled group are presented in Fig. 4. The overall median PFS was 7.2 months (95% CI, 4.2–9.1) in 3 L treatment, while the median rwPFS in the EGFRm subgroup was 7.1 months (95% CI, 4.1–9.1) (Fig. 4). Among all regimens, T+ showed a median rwPFS of 12.0 months (95% CI, 5.9-NE) in 3 L treatment (Fig. 4A).

### rwTTD and rwTTNT

The outcomes of rwTTD and rwTTNT in both the 2 L- and 3 L-enrolled groups are presented in Supplementary Fig. 3.

In the 2 L-enrolled group, patients had a median rwTTD of 7.0 months (95% CI, 6.2–7.7), as shown in Supplementary Fig. 3 and Table 7. Patients with EGFR mutations had a median rwTTD of 7.1 months (95% CI, 6.2–7.7) (Supplementary Fig. 3B), while those without EGFR mutations had a shorter median rwTTD of 6.3 months (95% CI, 4.5–10.2) (Supplementary Fig. 3C). The median rwTTNT was 9.0 months (95% CI, 8.0–10.0) (Supplementary Fig. 3D and Table 8). Among EGFR-mutated patients, the median rwTTNT was slightly shorter at 8.9 months (95% CI, 8.0–10.0) (Supplementary Fig. 3E), whereas non-EGFR-mutated patients demonstrated a longer median rwTTNT of 9.5 months (95% CI, 5.8–23.7) (Supplementary Fig. 3F).

## Discussion

This study provides a comprehensive overview of real-world treatment patterns, biomarker testing, and clinical outcomes in Chinese patients with advanced NSCLC harboring AGAs. While targeted monotherapy remained the predominant 2 L approach, the median rwPFS for the 2 L setting was 7.4 months, with targeted therapies achieving a median rwPFS of 9.4 months. Specifically, the EGFR-mutant subgroup achieved a median rwPFS of 7.5 in the 2 L setting, demonstrating sustained effectiveness. Furthermore, rwTTD and rwTTNT closely paralleled rwPFS, reinforcing the durability of current therapeutic pathways. It should be emphasized that the present AGA cohort was overwhelmingly composed of EGFR‑mutant cases (approximately 90%), and the most granular analyses (e.g. treatment sequences) focused on this subgroup; consequently, the reported treatment patterns and outcomes primarily reflect real‑world management of EGFR‑mutant NSCLC.

Interpretation of these treatment sequences must also consider the regulatory and reimbursement context during the study period (Supplementary materials). Patients were enrolled for second- or third-line treatment between September 2019 and December 2022, a period characterized by broad routine use of first- and second-generation EGFR-TKIs and rapid expansion of third-generation EGFR-TKI approval and NRDL coverage in China. Accordingly, the line-specific patterns depicted in Figs. 1 and 2 likely reflect a combination of disease biology, resistance mechanisms, and evolving access to reimbursed agents; for example, the predominance of earlier-generation TKIs in 1 L and the high use of targeted monotherapy in T790M-positive patients in 2 L are consistent with contemporaneous availability in Chinese practice. Unlike randomized controlled trials with restrictive eligibility criteria, this multicenter real-world cohort included a broad spectrum of patients, including those with comorbidities, prior therapies, and progression patterns (such as oligoprogression and CNS involvement) that often preclude trial participation. As a result, the observed patterns reflect guideline-based recommendations layered onto pragmatic considerations such as patient fitness, local practice norms, and changing drug accessibility.

The reliability of the real-world outcomes is underpinned by comprehensive baseline molecular profiling. Between 2019 and 2022, all patients in the screening cohort underwent molecular testing; EGFR was the most frequently assessed biomarker (90.9%), and 89.7% of the cohort harbored EGFR mutations. Testing for other actionable targets was also robust, including ALK (15.2%), ROS1 (8.8%), MET (6.8%), BRAF (5.3%), and RET (3.2%), with corresponding positivity rates of 6.7%, 2.0%, 4.3%, 2.0%, and 1.1%. Notably, even for ultra-rare targets such as NTRK, 1.5% of patients underwent testing, all of whom were negative. These patterns reflect growing adherence to guideline recommendations for broad molecular profiling and the increasing uptake of multigene NGS panels in Chinese practice [23–25]. The observed positivity rates are closely aligned with established Chinese epidemiologic data (MET: 0.9–5.0% [26, 27]; BRAF: 2.8% [28]; ROS1: 2.6% [29]; RET: 1.3% [30]), and with recent large-scale real-world NGS cohorts reporting similar ranges for ALK, MET, BRAF, ROS1, and RET [5, 31]. The concordance between our data and prior literature indicates that the molecular characteristics of this cohort are broadly representative of the wider Chinese NSCLC population, reinforcing the credibility of subsequent outcome analyses.

These comprehensive molecular profiles directly inform management of acquired resistance, particularly in navigating the increasingly complex EGFR-TKI sequencing landscape. Regarding later-line selection, the study reveals a notable divergence between real-world practice and guideline recommendations, especially around EGFR-TKI rechallenge. Although guidelines advocate platinum-based chemotherapy after progression on first-line TKIs in T790M-negative patients or on second-line third-generation TKIs [6, 7], detailed sequencing in our EGFR-mutant subgroup highlights the central role of continued TKI use across resistance phenotypes. This trend persists regardless of whether TKI monotherapy or combination (T+) was employed, and appears highly adaptive to initial treatment context: patients failing 1 L third-generation TKIs frequently received TKI-based combinations (T+) as 2 L regimens, while those failing 1 L first/second-generation TKIs without T790M often continued TKI monotherapy into 2 L and even 3 L. Even among T790M-positive patients who had already received 2 L third-generation TKIs, many continued third-generation TKI rechallenge as 3 L therapy. This persistent utilization underscores both the indispensable status of EGFR-TKIs and the current therapeutic predicament following acquired resistance; in the absence of optimal alternatives, TKI rechallenge represents a pragmatic “forced choice” [32], supported by the biological plausibility that the original EGFR driver may remain oncogenic despite subclonal heterogeneity [33, 34]. Extended TKI use may be particularly justified in indolent disease or oligoprogression, which are estimated to occur in 15–47% of NSCLC cases [35, 36]. A Phase II trial of local ablative therapy followed by osimertinib rechallenge in oligoprogressive EGFR-mutant NSCLC has preliminarily validated the safety of this strategy (median PFS 3.7 months) [37]. Together with emerging data from ORCHARD [38], TROPION-Lung15 [39], and sacituzumab tirumotecan (SKB264) [40], these findings suggest that TKI rechallenge, currently a pragmatic solution, may evolve into a more formalized, evidence-based therapeutic pillar. In parallel, the frequent later-line use of TKI continuation or rechallenge, especially in oligoprogression, reflects a real-world strategy of maintaining a partially effective TKI backbone while addressing progressing lesions with local or additional systemic therapies. This approach balances biological plausibility with the need to preserve tolerability in patients often unfit for intensive chemotherapy, yet remains largely driven by physician judgment and individual circumstances.

In routine practice, post-first-line treatment selection is highly individualized. Baseline ECOG performance status, comorbidities, and subsequent functional changes influence whether patients receive combination regimens, cytotoxic chemotherapy, or more conservative options. Patterns of progression, such as oligoprogression or isolated CNS progression, often prompt continuation of an effective TKI backbone with local therapies or targeted combinations rather than immediate escalation to chemotherapy. Physician preference, institutional experience, and evolving reimbursement and formulary status for third-generation EGFR-TKIs and anti-angiogenic agents also likely contributed to the heterogeneity observed in later-line regimens. Because these factors were neither prospectively standardized nor formally modeled, the reported sequences should be interpreted as descriptive of real-world decision-making, rather than as prescriptive algorithms.

The RECAP study showed a median rwPFS of 7.4 months for second-line treatment in Chinese patients with advanced NSCLC harboring AGAs. When stratified by modality, survival outcomes display nuanced consistencies and deviations relative to established literature: conventional chemotherapy (C) and combination chemotherapy (C+) yielded median 2 L rwPFS of 4.2 and 6.3 months, respectively, in line with prior reports of standard chemotherapy (4.2 months) and chemoimmunotherapy (6.7 months) post–EGFR-TKI failure [41], as well as platinum-doublet outcomes (~ 6.2 months) in similar refractory cohorts [42]. TKI-based strategies showed numerically longer rwPFS; in the overall 2 L setting, targeted monotherapy (T) and targeted combinations (T+) achieved median rwPFS of 7.7 and 9.4 months, respectively, representing the numerically highest values among observed modalities. For patients progressing on 1 L first- or second-generation TKIs, historical data indicate that 2 L osimertinib yields PFS of ~ 11.2 months in carefully selected T790M-positive cohorts versus ~3.1 months in T790M-negative patients [43]. In our corresponding real-world subgroup, 2 L T and T+ strategies produced median rwPFS of 7.6 and 8.7 months, respectively, reflecting the more heterogeneous resistance profiles typical of unselected populations [15, 16]. Prior observational studies of continuous EGFR-TKI beyond progression reported a median extended benefit of ~ 5.1 months [32], underscoring that survival gains in unselected, biologically diverse cohorts are inherently more modest than in tightly biomarker-directed settings. A distinct pattern emerged among patients progressing on 1 L third-generation TKIs: in this increasingly common and therapeutically challenging scenario, where established targeted options remain limited, those receiving 2 L T+ regimens achieved a median rwPFS of 14.0 months. While this numerically extended rwPFS suggests potential benefit from maintaining a third‑generation TKI backbone in selected patients, these findings derive from a small, retrospectively identified subgroup and should be viewed as hypothesis‑generating. Differences in baseline risk, disease biology, and physician preference may contribute to the observed outcomes, and no formal comparative analyses were conducted to establish superiority over alternatives. Consistent signals from ALTER-L058 [44], smaller retrospective series [45], and the COMPEL trial [46] indicate that continuing a third‑generation TKI with an additional mechanism‑based agent can yield clinically meaningful disease control beyond initial progression. The numerically longer rwPFS observed in our cohort may reflect regimen heterogeneity (various TKI-anti-angiogenic or TKI-chemotherapy combinations) and real-world selection bias favoring fitter or more indolent cases, as well as broader differences in demographics and resistance patterns. Overall, these subgroup findings suggest that, in carefully selected patients, continued third‑generation TKI-based combinations or anti-angiogenic-based regimens can numerically prolong disease control in practice, but small sample sizes, wide confidence intervals, and a retrospective design preclude claims of strategy superiority and instead highlight approaches warranting prospective evaluation.

Notably, a very small subset of EGFR‑mutated patients (*n* = 11) receiving 2 L anti‑angiogenic monotherapy (A) exhibited a numerically prolonged median rwPFS of 18.4 months (95% CI, 2.9-NE). Given the limited sample size and wide confidence interval, this finding should be interpreted as exploratory and hypothesis‑generating rather than indicative of the superiority of anti‑angiogenic monotherapy over other options. Such prolonged benefit is likely due to substantial selection bias, as early-line single-agent therapy is often reserved for patients with inherently indolent disease trajectories. Despite this striking outlier, the study also provides insight into chemotherapy-free salvage options in heavily pretreated populations. In the 3 L setting and beyond, anti-angiogenic monotherapy achieved a median rwPFS of 4.7 months, consistent with prior real-world and retrospective Chinese data showing median PFS of ~ 4.0–5.6 months for apatinib or anlotinib in refractory NSCLC [17, 44]. Given cumulative toxicities from prior therapies, anti-angiogenic monotherapy represents a pragmatic “safety net,” offering modest but stable disease control for patients ineligible for intensive cytotoxic regimens. In the subgroup developing new brain metastases during 2 L therapy, a median rwPFS of 7.3 months suggests that clinicians may preferentially select regimens with perceived CNS activity, potentially including third-generation EGFR-TKIs when feasible, consistent with trial data showing CNS efficacy of osimertinib in EGFR-mutant NSCLC with brain metastases. However, because TKI generation was not systematically recorded for all patients in this subgroup, these observations should be regarded as hypothesis-generating rather than proof of regimen-specific CNS benefit.

A strength of this study is its conduct across six leading tertiary hospitals with relatively standardized molecular testing and rapid adoption of newly approved and reimbursed anticancer therapies, which reduces the likelihood that local stockouts or idiosyncratic access barriers drive observed sequencing patterns and increases the likelihood that they reflect broader national approval and reimbursement trends. Nonetheless, several limitations must be acknowledged. The retrospective design introduces potential selection bias, and substantial missingness and heterogeneity in safety data represent a major constraint. Although adverse events were abstracted and graded according to CTCAE when documented, low‑grade toxicities, dose adjustments, and interruptions were inconsistently recorded, and only a subset of patients had sufficiently complete safety information. To avoid biased or non‑representative summaries, detailed regimen‑specific safety comparisons were not performed. It is particularly relevant in later‑line NSCLC, where sequencing decisions often hinge on trade‑offs between modest efficacy gains, cumulative toxicity, and comorbidities; thus, the reported trajectories should be interpreted primarily in terms of effectiveness, with the understanding that unmeasured tolerability considerations likely influenced real-world choices. Future real-world studies should prospectively incorporate structured safety assessments and patient-reported tolerability measures to better support patient-centered sequencing. In addition, detailed longitudinal data on performance status at each line, progression patterns (e.g., oligoprogression vs. diffuse progression), CNS involvement, and physician rationale were not systematically recorded. Consequently, the relative contributions of patient fitness, progression pattern, physician preference, and drug accessibility to observed choices cannot be disentangled, and the sequences described here remain descriptive rather than prescriptive. The predominance of EGFR‑mutated patients limits generalizability to other AGAs; small sample sizes for non‑EGFR alterations (e.g., ALK, ROS1, RET, MET, BRAF) preclude robust subtype-specific inferences, and the detailed patterns and outcomes should be considered primarily representative of EGFR‑mutant NSCLC. Real-world OS could not be estimated due to the low number of death events (1.5%, 10/658) during follow-up, so long-term survival benefits of these multi-line strategies remain uncertain. Progression events were ascertained from routine clinical documentation and predominantly reflect physician‑assessed progression, which may not strictly follow RECIST in all cases and represents an inherent limitation of retrospective rwPFS estimation. Several subgroup analyses, including those evaluating anti‑angiogenic monotherapy and TKI-based combinations after third‑generation EGFR-TKIs, involved very small numbers and are highly susceptible to selection bias and residual confounding; the study was neither designed nor powered for robust comparative effectiveness analyses. Finally, incomplete capture of post‑2 L biomarker testing prevented comprehensive analysis of dynamic molecular evolution and formal linkage of specific resistance mechanisms to 3 L and later choices, meaning the observed sequences likely reflect a mix of biologically informed and empiric decisions. The absence of patient-reported outcomes or health-related quality-of-life measures further limits assessment of symptom burden, functional status, and tolerability from the patient perspective, which is increasingly relevant in later-line oncology. Future real-world studies should therefore integrate systematic molecular, safety, and PRO data to support more mechanistic and patient-centered optimization of treatment sequencing [47].

## Conclusion

The RECAP study highlights substantial therapeutic heterogeneity and evolving treatment paradigms for advanced AGA‑driven NSCLC in real‑world Chinese practice, particularly within the predominant EGFR‑mutant population. Given the limited numbers of non‑EGFR AGA cases, the detailed treatment‑sequencing patterns and outcome estimates presented here should be considered most representative of EGFR‑mutant NSCLC, with extrapolation to other oncogenic drivers requiring further dedicated studies. Targeted therapies, particularly adaptive TKI continuation or combination strategies, remain the cornerstone of later-line management, offering pragmatic survival benefits. Notably, although molecular profiling upon disease progression is widely adopted to elucidate resistance mechanisms, the frequent reliance on TKI rechallenge or combinations highlights a critical therapeutic gap when actionable secondary targets are absent or standard options are exhausted. As survival outcomes inevitably plateau in these refractory settings, integrating precise biomarker guidance with emerging novel therapeutics will be essential to overcome current efficacy bottlenecks and reshape future treatment sequencing.

## Supplementary Information

Below is the link to the electronic supplementary material.

Supplementary Material 1

## Data Availability

The data that support the findings of this study are available on request from the corresponding author.
