# Supplementary material for: Treatment patterns and outcomes of second/third-line therapy in advanced non-small cell lung cancer with actionable genomic alterations (RECAP)
Source: Clin Exp Metastasis. 2026 Jul 30;43(4):38. doi: 10.1007/s10585-026-10417-x (PMC13423968; doi:10.1007/s10585-026-10417-x)

**Supplementary Materials**

**1 Supplementary Text**

**Section 1. The definition of effectiveness metrics in the real-world setting.**

Real-world progression-free survival (rwPFS) was defined as the interval from the initiation of the indexed treatment line to the earliest occurrence of documented disease progression, death from any cause, or initiation of the subsequent treatment line when the date of progression was unavailable. Patients without a recorded event were censored at the date of the last administration of lung cancer-specific therapy or the initiation of a new anti-tumor regimen, whichever came first. Real-world time to treatment discontinuation (rwTTD) was measured from the start of each treatment line to the earliest of documented disease progression, treatment discontinuation, or death. For patients who remained on therapy without recorded discontinuation or death during the observation period, rwTTD was censored at the last date of lung cancer-related medication for that treatment line. Real-world time to next treatment or death (TTNT) was defined as the time from the initiation of the current therapy to the start date of the next systemic treatment. Patients without subsequent therapy or with missing treatment start dates were censored at the last available medical record entry. Real-world overall survival (rwOS) was calculated from the date of diagnosis of advanced non-small cell lung cancer (NSCLC) to the date of death from any cause. For patients alive at the end of follow-up or with no record of death, rwOS was censored at the date of the most recent medical documentation reflecting known survival status.

**Section 2. Regulatory Approval and Reimbursement Landscape of Target Therapies**

In real-world practice in China, the adoption of EGFR-TKIs across different lines of therapy has been strongly shaped by three interrelated factors: (i) the regulatory approval of first-, second-, and third-generation TKIs, (ii) their inclusion in and timing of updates to the National Reimbursement Drug List (NRDL), and (iii) institutional drug availability. Our study enrolled patients with advanced/metastatic NSCLC who initiated second- or third-line systemic therapies between September 1, 2019, and December 31, 2022, with follow-up censored on October 30, 2023. This observation window overlaps with broad NRDL coverage of first- and second-generation EGFR-TKIs, as well as the rapid market uptake and reimbursement integration of third-generation EGFR-TKIs (e.g. osimertinib, almonertinib, and furmonertinib). Accordingly, the evolving treatment sequences illustrated in our stacked bar chart (Figure 1) and Sankey diagrams (Figure 2) are closely aligned with these policy and access dynamics.

First, for first-line therapy administered prior to study enrollment, early-generation TKIs were the predominant choice, reflecting both their earlier integration into the NRDL and prevailing clinical practice patterns at that prior time. Although osimertinib obtained first-line approval in August 2019, NRDL reimbursement for first-line osimertinib was not implemented until March 2021, and first-line NRDL coverage for domestic third-generation TKIs (almonertinib and furmonertinib) only came into effect in 2023. During the earlier years of our enrollment period, third-generation TKIs therefore remained relatively less affordable and less widely adopted in the frontline setting. In addition, many Chinese oncologists remained cautious about using third-generation TKIs upfront, given ongoing debate about whether to reserve the “best” drug for later lines versus deploying it in first line in the absence of conclusive overall survival advantages between sequencing strategies. As a result, among patients with EGFR-mutated NSCLC in our cohort, most received first- or second-generation TKIs as first-line therapy, with a gradual increase in the use of third-generation TKIs toward the latter part of the study. This temporal transition is captured in Figure 2A, where a notable subgroup of 136 patients received third-generation TKIs as first-line treatment.

Second, in the second-line setting, drug accessibility was already high for third-generation TKIs targeting T790M-positive disease. Osimertinib was approved for second-line use in March 2017 and had NRDL reimbursement implemented in October 2018. Thus, at the start of our study (September 2019), second-line osimertinib was widely available and affordable in major Chinese hospitals. This context explains why, in the T790M-positive subgroup (Figure 2B), 88.5% of patients received targeted monotherapy as their second-line regimen. During the study period, the therapeutic landscape was further enriched by the introduction and subsequent NRDL inclusion of domestic third-generation TKIs (almonertinib and furmonertinib) for second-line use, with reimbursement implementation occurring in 2021 and 2022, respectively. These sequential regulatory and reimbursement milestones substantially expanded the second-line targeted therapy options for T790M-positive patients in routine practice.

Third, the third-line setting was strongly conditioned by prior exposure to targeted therapy in earlier lines. Because most patients had already undergone multiple lines of targeted therapy, third-line treatment patterns reflected a shift toward salvage strategies rather than de novo adoption of novel targeted agents. As exemplified in Figure 2, among patients receiving third-line therapy, platinum-based chemotherapy combinations (designated as C+) were the most frequently adopted regimen, with proportions ranging from 16.9% to 42.6% across different EGFR-mutated subgroups, although a subset of patients continued cross-line TKI (designated as T/T+) as their third-line treatment. This pattern underscores that third-line therapeutic decisions are not made in isolation; instead, they represent downstream consequences of high upstream accessibility to targeted agents and the cumulative exhaustion of available EGFR-TKI options.

Finally, we emphasize that the RECAP study was conducted across six national Class A tertiary hospitals in China, which are leading institutions with highly synchronized processes for introducing new drugs and implementing NRDL policies. This institutional homogeneity, coupled with the nationwide NRDL framework, mitigates the risk that the observed treatment patterns were driven by sporadic “drug stockouts” or local unavailability at individual centers. Rather, the treatment trajectories observed in our cohort can be regarded as a representative microcosm of how macro-level regulatory approvals, reimbursement policies, and guideline updates collectively shape real-world sequencing of targeted therapies in Chinese patients with advanced NSCLC.

**2 Supplementary Tables and Figures**

**Supplementary Table 1. Biomarker testing pattern.**

| Variable, *n* (%) | All (N = 658) |
| --- | --- |
| EGFR alteration record | 598 (90.9) |
| Positive | 590 (89.7) |
| Negative | 8 (1.2) |
| ALK alteration record | 100 (15.2) |
| Positive | 44 (6.7) |
| Negative | 56 (8.5) |
| ROS1 alteration record | 58 (8.8) |
| Positive | 13 (2.0) |
| Negative | 45 (6.8) |
| NTRK alteration record | 10 (1.5) |
| Positive | 0 |
| Negative | 10 (1.5) |
| BRAF alteration record | 35 (5.3) |
| Positive | 13 (2.0) |
| Negative | 22 (3.3) |
| RET alteration record | 21 (3.2) |
| Positive | 7 (1.1) |
| Negative | 14 (2.1) |
| MET alteration record | 45 (6.8) |
| Positive | 28 (4.3) |
| Negative | 17 (2.6) |

EGFR, epidermal growth factor receptor; ALK, ALK receptor tyrosine kinase; ROS1, ROS proto-oncogene 1; NTRK, neurotrophic receptor tyrosine kinase; BRAF, B-Raf proto-oncogene, serine/threonine kinase; RET, ret proto-oncogene; MET, MET proto-oncogene, receptor tyrosine kinase.

**Supplementary Table 2. Distribution of treatments across therapy lines in the 2L- and 3L-enrolled groups.**

| Treatment | First-line | Second-line | Third-line | Fourth-line |
| --- | --- | --- | --- | --- |
| 2L-enrolled patients | N=602 | N=602 | N=404 | N=182 |
| T | 453 (75.3) | 297 (49.3) | 79 (19.6) | 41 (22.5) |
| T+C | 77 (12.8) | 24 (4.0) | 14 (3.5) | 1 (0.6) |
| T+A+C | 37 (6.2) | 23 (3.8) | 12 (3.0) | 3 (1.6) |
| T+A | 34 (5.7) | 31 (5.2) | 26 (6.4) | 14 (7.7) |
| I+A+C | 1 (0.2) | 16 (2.7) | 26 (6.4) | 10 (5.5) |
| I+C | 0 | 23 (3.8) | 27 (6.7) | 13 (7.1) |
| C | 0 | 60 (10.0) | 49 (12.1) | 29 (15.9) |
| A+C | 0 | 99 (16.5) | 109 (27.0) | 35 (19.2) |
| I | 0 | 8 (1.3) | 5 (1.2) | 4 (2.2) |
| I+A | 0 | 5 (0.8) | 8 (2.0) | 4 (2.2) |
| T+I+C | 0 | 0 | 3 (0.7) | 2 (1.1) |
| A | 0 | 16 (2.7) | 45 (11.1) | 26 (14.3) |
| T+I | 0 | 0 | 1 (0.3) | 0 |
| 3L-enrolled patients | N=56 | N=56 | N=56 | N=30 |
| T | 42 (75.0) | 48 (85.7) | 6 (10.7) | 12 (40.0) |
| T+C | 2 (3.6) | 2 (3.6) | 2 (3.6) | 1 (3.3) |
| T+A+C | 0 | 1 (1.8) | 2 (3.6) | 1 (3.3) |
| T+A | 1 (1.8) | 4 (7.1) | 4 (7.1) | 5 (16.7) |
| I+A+C | 2 (3.6) | 0 | 2 (3.6) | 1 (3.3) |
| I+C | 2 (3.6) | 0 | 3 (5.4) | 0 |
| C | 0 | 0 | 8 (14.3) | 2 (6.7) |
| A+C | 5 (8.9) | 1 (1.8) | 20 (35.7) | 2 (6.7) |
| I | 0 | 0 | 1 (1.8) | 2 (6.7) |
| I+A | 0 | 0 | 1 (1.8) | 2 (6.7) |
| A | 2 (3.6) | 0 | 7 (12.5) | 2 (6.7) |

T: targeted therapy; I: immunotherapy; A: anti-angiogenic therapy; C: chemotherapy.

**Supplementary Table 3. Treatment distributions stratified by therapy line within EGFR-mutant (EGFRm) and non-EGFR mutant (non-EGFRm) subgroups of the 2L-enrolled and 3L-enrolled groups.**

| Treatment | First-line | | Second-line | | Third-line | | Fourth-line | |
| --- | --- | --- | --- | --- | --- | --- | --- | --- |
|  | EGFRm | Non-EGFRm | EGFRm | Non-EGFRm | EGFRm | Non-EGFRm | EGFRm | Non-EGFRm |
| 2L-enrolled patients | N=542 | N=60 | N=542 | N=60 | N=372 | N=32 | N=164 | N=18 |
| T | 404 (74.5) | 49 (81.67) | 264(48.7) | 33(55.0) | 66(17.7) | 13(40.6) | 38(23.2) | 3(16.7) |
| T+C | 73 (13.6) | 4 (6.67) | 22(4.1) | 2(3.3) | 13(3.5) | 1(3.1) | 1(0.6) | 0 |
| T+A+C | 32 (5.9) | 5 (8.33) | 20(3.7) | 3(5.0) | 12(3.2) | 0 | 3(1.8) | 0 |
| T+A | 32 (5.9) | 2 (3.33) | 29(5.4) | 2(3.3) | 25(6.7) | 1(3.1) | 14(8.5) | 0 |
| I+A+C | 1 (0.2) | 0 | 14(2.6) | 0 | 26(7.0) | 0 | 10(6.1) | 0 |
| A+C | 0 | 0 | 91(16.8) | 8(13.3) | 98(26.3) | 11(34.4) | 32(19.5) | 3(16.7) |
| C | 0 | 0 | 56(10.3) | 4(6.7) | 48(12.9) | 1(3.1) | 23(14.0) | 6(33.3) |
| I+C | 0 | 0 | 19(3.5) | 4(6.7) | 25(6.7) | 2(6.3) | 10(6.1) | 3(16.7) |
| A | 0 | 0 | 16(3.0) | 0 | 42(11.3) | 3(9.4) | 25(15.2) | 1(5.6) |
| I | 0 | 0 | 6(1.1) | 2(3.3) | 5(1.3) | 0 | 2(1.2) | 2(11.1) |
| A+I | 0 | 0 | 5(0.9) | 0 | 8(2.2) | 0 | 4(2.4) | 0 |
| I+T+C | 0 | 0 | 0 | 0 | 3(0.8) | 0 | 2(1.2) | 0 |
| I+T | 0 | 0 | 0 | 0 | 1(0.3) | 0 | 0 | 0 |
| 3L-enrolled patients | N=48 | N=8 | N=48 | N=8 | N=48 | N=8 | N=28 | N=2 |
| T | 40 (83.3) | 2 (25.0) | 40 (83.3) | 8 (100.0) | 6(12.5) | 0 | 10(35.7) | 2(100.0) |
| A+C | 5 (10.4) | 0 | 1 (2.1) | 0 | 16(33.3) | 4(50.0) | 2(7.1) | 0 |
| T+C | 1 (2.1) | 1 (12.5) | 2 (4.2) | 0 | 2(4.2) | 0 | 1(3.6) | 0 |
| A | 1 (2.1) | 1 (12.5) | 1 (2.1) | 0 | 4(8.3) | 3(37.5) | 2(7.1) | 0 |
| I+A+C | 0 | 2 (25.0) | 0 | 0 | 2(4.2) | 0 | 1(3.6) | 0 |
| I+C | 0 | 2 (25.0) | 0 | 0 | 3(6.3) | 0 | 0 | 0 |
| A+T | 1 (2.1) | 0 | 4 (8.3) | 0 | 4(8.3) | 0 | 5(17.9) | 0 |
| C | 0 | 0 | 0 | 0 | 8(16.7) | 0 | 2(7.1) | 0 |
| A+T+C | 0 | 0 | 0 | 0 | 2(4.2) | 0 | 1(3.6) | 0 |
| A+I | 0 | 0 | 0 | 0 | 1(2.1) | 0 | 2(7.1) | 0 |
| I | 0 | 0 | 0 | 0 | 0 | 1(12.5) | 2(7.1) | 0 |

2L: second-line; 3L: third-line; T: targeted therapy; I: immunotherapy; A: anti-angiogenic therapy; C: chemotherapy.

**Supplementary Table 4. Therapy distributions at 2L in 2L-enrolled patients receiving platinum-based chemotherapy in 2L therapy**.

| Treatment | Non-platinum chemotherapy in second-line therapy (N=408) | Platinum-based chemotherapy in second-line therapy (N=194) |
| --- | --- | --- |
| T | 297 (72.8) | 0 |
| T+C | 7 (1.7) | 17 (8.8) |
| T+A+C | 9 (2.2) | 14 (7.2) |
| T+A | 31 (7.6) | 0 |
| I+A+C | 2 (0.5) | 14 (7.2) |
| I+C | 5 (1.2) | 18 (9.3) |
| C | 9 (2.2) | 51 (26.3) |
| A+C | 19 (4.7) | 80 (41.2) |
| I | 8 (2.0) | 0 |
| I+A | 5 (1.2) | 0 |
| A | 16 (3.9) | 0 |

2L: second-line; 3L: third-line; T: targeted therapy; I: immunotherapy; A: anti-angiogenic therapy; C: chemotherapy.

**Supplementary Table 5. Therapy distributions at 2L and post-progression 3L in 2L-enrolled patients with new brain metastases**

| Treatment | Second-line  N=46 | Third-line  N=2 |
| --- | --- | --- |
| T | 37 (80.4) | 0 |
| T+C | 3 (6.5) | 0 |
| T+A+C | 2 (4.4) | 0 |
| A+C | 1 (2.2) | 0 |
| A+T | 1 (2.2) | 0 |
| C | 1 (2.2) | 0 |
| A+I+C | 1 (2.2) |  |
| A+C | 0 | 2 (100.0) |

2L: second-line; 3L: third-line; T: targeted therapy; I: immunotherapy; A: anti-angiogenic therapy; C: chemotherapy.

**Supplementary Table 6. Disease progression and real-world progression-free survival (rwPFS) within the 2L-enrolled and 3L-enrolled groups.**

| Patients | Treatment lines | Treatment  regimens | n | Progression, n (%) | Death, n (%) | Median rwPFS (95% CI), months |
| --- | --- | --- | --- | --- | --- | --- |
| 2L-enrolled patients | 2L | Total | 602 | 420 (69.8) | 3 (0.5) | 7.4 (6.5, 8.0) |
|  |  | T | 297 | 238 (80.1) | 0 | 7.7 (6.8, 9.2) |
|  |  | A+C | 99 | 62 (62.6) | 0 | 6.3 (5.8, 7.9) |
|  |  | C | 60 | 38 (63.3) | 1 (1.7) | 4.2 (3.5, 5.8) |
|  |  | T+A | 31 | 23 (74.2) | 0 | 7.0 (4.4, 12.3) |
|  |  | T+C | 24 | 12 (50.0) | 0 | 9.4 (7.7, NE) |
|  |  | I+C | 23 | 13 (56.5) | 1 (4.4) | 5.9 (3.7, NE) |
|  |  | T+A+C | 23 | 14 (60.9) | 0 | 9.7 (7.5, NE) |
|  |  | A | 16 | 4 (25.0) | 0 | 7.0 (2.9, NE) |
|  |  | I+A+C | 16 | 10 (62.5) | 0 | 7.9 (5.1, NE) |
|  |  | I | 8 | 4 (50.0) | 1 (12.5) | 3.9 (1.3, NE) |
|  |  | I+A | 5 | 2 (40.0) | 0 | 12.6 (4.3, NE) |
| 3L-enrolled patients | 3L | Total | 56 | 33 (58.9) | 1 (1.8) | 7.2 (4.2, 9.1) |
|  |  | A+C | 20 | 10 (50.0) | 0 | 8.4 (7.1, NE) |
|  |  | C | 8 | 6 (75.0) | 0 | 3.6 (1.9, NE) |
|  |  | A | 7 | 2 (28.6) | 1 (14.3) | 10.7 (0.7, NE) |
|  |  | T | 6 | 5 (83.3) | 0 | 3.3 (2.5, NE) |
|  |  | T+A | 4 | 3 (75.0) | 0 | 19.5 (15.0, NE) |
|  |  | I+C | 3 | 2 (66.7) | 0 | 13.8 (9.1, NE) |
|  |  | I+A+C | 2 | 1 (50.0) | 0 | 4.0 (NE, NE) |
|  |  | T+A+C | 2 | 2 (100.0) | 0 | 8.9 (5.9, NE) |
|  |  | T+C | 2 | 2 (100.0) | 0 | 5.7 (3.7, NE) |
|  |  | I | 1 | 0 | 0 | NE (NE, NE) |
|  |  | I+A | 1 | 0 | 0 | NE (NE, NE) |

2L: second-line; 3L: third-line; T: targeted therapy; I: immunotherapy; A: anti-angiogenic therapy; C: chemotherapy; NE, not estimable; CI, confidence interval.

**Supplementary Table 7. Real-world time to treatment discontinuation (rwTTD) within the 2L-enrolled and 3L-enrolled groups**

| Patients | Treatment lines | Treatment  regimens | n | Progression, n (%) | Treatment discontinuation, n (%) | Death, n (%) | Median mTTD  (95% CI), months |
| --- | --- | --- | --- | --- | --- | --- | --- |
| 2L-enrolled patients | 2L | Total | 602 | 420 (69.8) | 17 (2.8) | 3 (0.5) | 7.0(6.2, 7.7) |
|  |  | T | 297 | 238 (80.1) | 5 (1.7) | 0 (0) | 7.6(6.7, 9.1) |
|  |  | A+C | 99 | 62 (62.6) | 2 (2.0) | 0 (0) | 6.3(5.8, 7.7) |
|  |  | C | 60 | 38 (63.3) | 5 (8.3) | 1 (1.7) | 3.6(2.6, 5.4) |
|  |  | A+T | 31 | 23 (74.2) | 2 (6.5) | 0 (0) | 6.4(4.0, 12.3) |
|  |  | T+C | 24 | 12 (50.0) | 1 (4.2) | 0 (0) | 8.8(7.7, -) |
|  |  | I+C | 23 | 13 (56.5) | 1 (4.4) | 1 (4.4) | 5.1(3.7, -) |
|  |  | A+T+C | 23 | 14 (60.9) | 0 (0) | 0 (0) | 9.7(7.5, -) |
|  |  | A | 16 | 4 (25.0) | 1 (6.3) | 0 (0) | 7.0(2.9, -) |
|  |  | A+I+C | 16 | 10 (62.5) | 0 (0) | 0 (0) | 7.9(5.1, -) |
|  |  | I | 8 | 4 (50) | 0 (0) | 1 (12.5) | 3.9(1.3, -) |
|  |  | A+I | 5 | 2 (40) | 0 (0) | 0 (0) | 12.6(4.3, -) |
| 3L-enrolled patients | 3L | Total | 56 | 33 (58.9) | 2 (3.6) | 1 (1.8) | 7.1(4.1, 8.7) |
|  |  | A+C | 20 | 10 (50.0) | 0 (0) | 0 (0) | 8.4(7.1, -) |
|  |  | C | 8 | 6 (75.0) | 1 (12.5) | 0 (0) | 2.2(1.9, -) |
|  |  | A | 7 | 2 (28.6) | 1 (14.3) | 1 (14.3) | 2.5(0.7, -) |
|  |  | T | 6 | 5 (83.3) | 0 (0) | 0 (0) | 3.3(2.5, -) |
|  |  | A+T | 4 | 3 (75.0) | 0 (0) | 0 (0) | 19.5(15.0, -) |
|  |  | I+C | 3 | 2 (66.7) | 0 (0) | 0 (0) | 13.8(9.1, -) |
|  |  | A+I+C | 2 | 1 (50.0) | 0 (0) | 0 (0) | 4.0(-, -) |
|  |  | A+T+C | 2 | 2 (100) | 0 (0) | 0 (0) | 8.9(5.9, -) |
|  |  | T+C | 2 | 2 (100) | 0 (0) | 0 (0) | 5.7(3.7, -) |
|  |  | I | 1 | 0 (0) | 0 (0) | 0 (0) | -(-, -) |
|  |  | A+I | 1 | 0 (0) | 0 (0) | 0 (0) | -(-, -) |

2L: second-line; 3L: third-line; T: targeted therapy; I: immunotherapy; A: anti-angiogenic therapy; C: chemotherapy.

**Supplementary Table 8. Real-world time to next treatment or death (rwTTNT) within the 2L-enrolled and 3L-enrolled groups.**

| Patients | Treatment lines | Treatment regimens | n | Next-line therapy, n (%) | Median mTTNT (95% CI), months |
| --- | --- | --- | --- | --- | --- |
| 2L-enrolled patients | 2L | Total | 602 | 404 (67.1) | 9.0 (8.0, 10.0) |
|  |  | T | 297 | 235 (79.1) | 9.1 (7.7, 10.2) |
|  |  | A+C | 99 | 52 (52.5) | 8.7 (7.2, 15.2) |
|  |  | C | 60 | 39 (65.0) | 5.4 (3.5, 9.3) |
|  |  | A+T | 31 | 23 (74.2) | 6.4 (4.6, 13.3) |
|  |  | T+C | 24 | 13 (54.2) | 9.5 (8.8, -) |
|  |  | I+C | 23 | 13 (56.5) | 9.7 (4.8, -) |
|  |  | A+T+C | 23 | 12 (52.2) | 17.8 (8.3, -) |
|  |  | A | 16 | 3 (18.8) | - (7.0, -) |
|  |  | A+I+C | 16 | 9 (56.3) | 14.5 (6.2, -) |
|  |  | I | 8 | 4 (50.0) | 4.4 (3.9, -) |
|  |  | A+I | 5 | 1 (20.0) | - (4.3, -) |
| 3L-enrolled patients | 3L | Total | 56 | 30 (53.6) | 9.1 (6.3, 18.4) |
|  |  | A+C | 20 | 7 (35.0) | 11.1 (8.4, -) |
|  |  | C | 8 | 5 (62.5) | 4.1 (3.6, -) |
|  |  | A | 7 | 3 (42.9) | 18.8 (4.8, -) |
|  |  | T | 6 | 5 (83.3) | 3.4 (2.5, -) |
|  |  | A+T | 4 | 3 (75.0) | 19.5 (15.0, -) |
|  |  | I+C | 3 | 2 (66.7) | 14.7 (11.0, -) |
|  |  | A+I+C | 2 | 1 (50.0) | 5.2 (4.0, -) |
|  |  | A+T+C | 2 | 2 (100) | 8.9 (5.9, -) |
|  |  | T+C | 2 | 2 (100) | 6.4 (3.7, -) |
|  |  | I | 1 | 0 (0) | - (-, -) |
|  |  | A+I | 1 | 0 (0) | - (-, -) |

2L: second-line; 3L: third-line; T: targeted therapy; I: immunotherapy; A: anti-angiogenic therapy; C: chemotherapy.

**Supplementary Figure 1. Patient flowchart.** NSCLC, non-small cell lung cancer; AGA, actionable genomic alteration; TKI, tyrosine kinase inhibitor; ICI, immune checkpoint inhibitor; 2L-enrolled group, patients were receiving second-line treatment at enrollment; 3L-enrolled group, patients were receiving third-line treatment at enrollment.


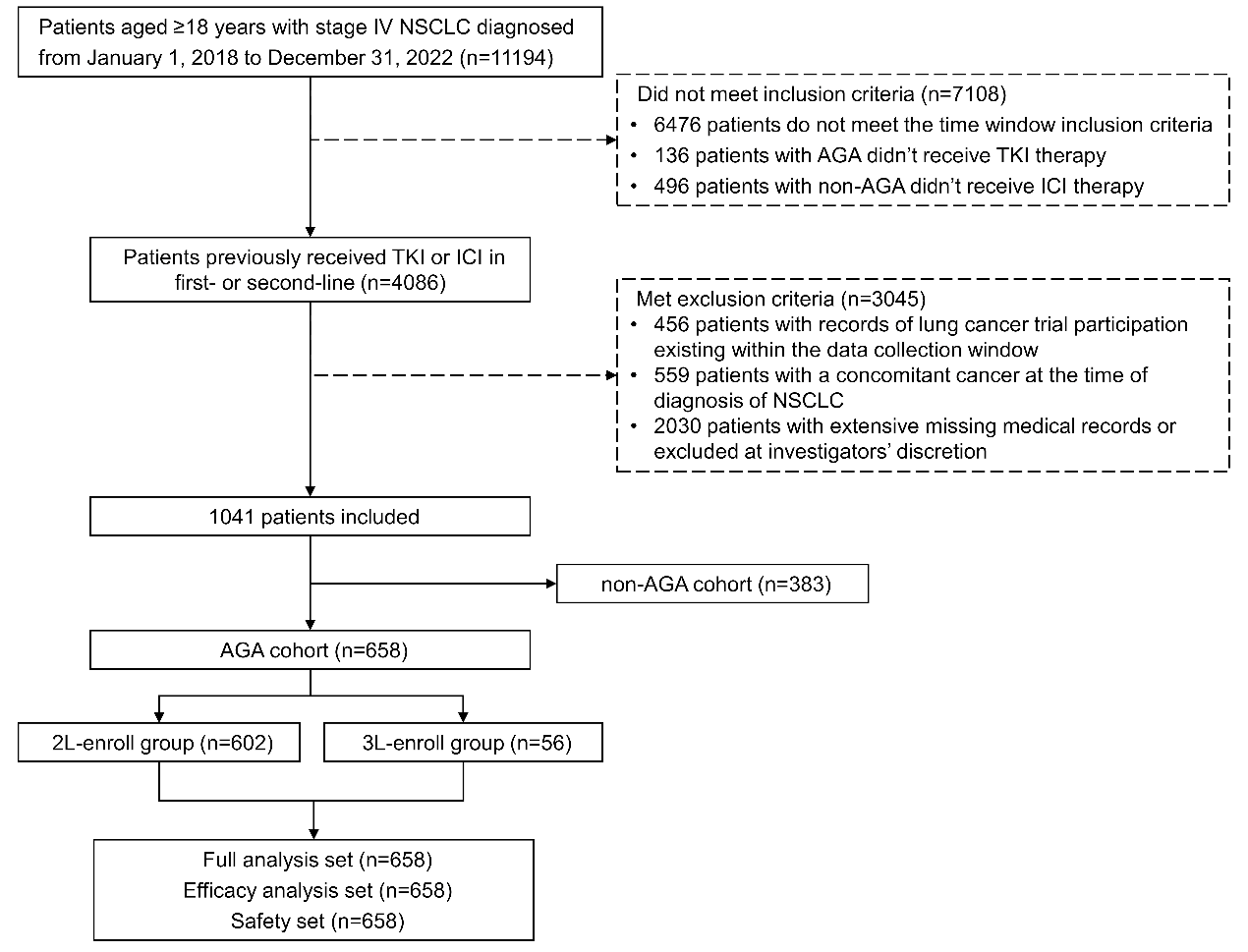


**Supplementary Figure 2. Real-world progression-free survival (rwPFS) in subgroups.** A) rwPFS in patients with newly developed brain metastases. B) rwPFS in patients with EGFR mutations. C) rwPFS in patients without EGFR mutations. D) rwPFS in second-line enrolled (2L-enrolled) patients receiving chemotherapy in 2L therapy stratified by platinum exposure.


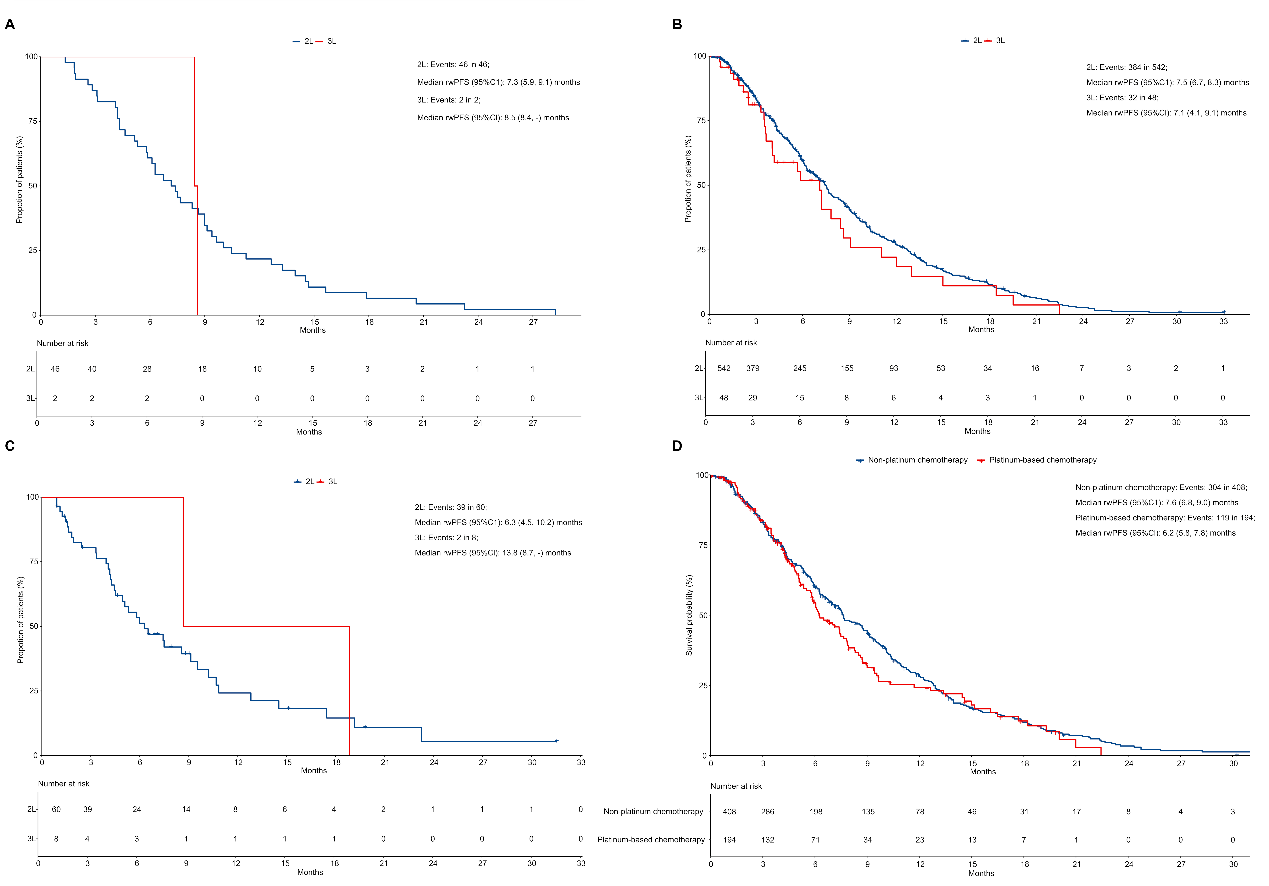


**Supplementary Figure 3. Overall rwTTD and rwTTNT.** A) rwTTD within 2L- and 3L- enrolled groups. B) rwTTD among patients with EGFR mutations. C) rwTTD among patients without EGFR mutations. D) rwTTNT within 2L- and 3L- enrolled cohorts. E) rwTTNT among patients with EGFR mutations. F) rwTTNT among patients without EGFR mutations. rwTTD, real-world time to treatment discontinuation; rwTTNT, time to next treatment or death; 2L, second line; 3L, third line; EGFRm, EGFR mutations.


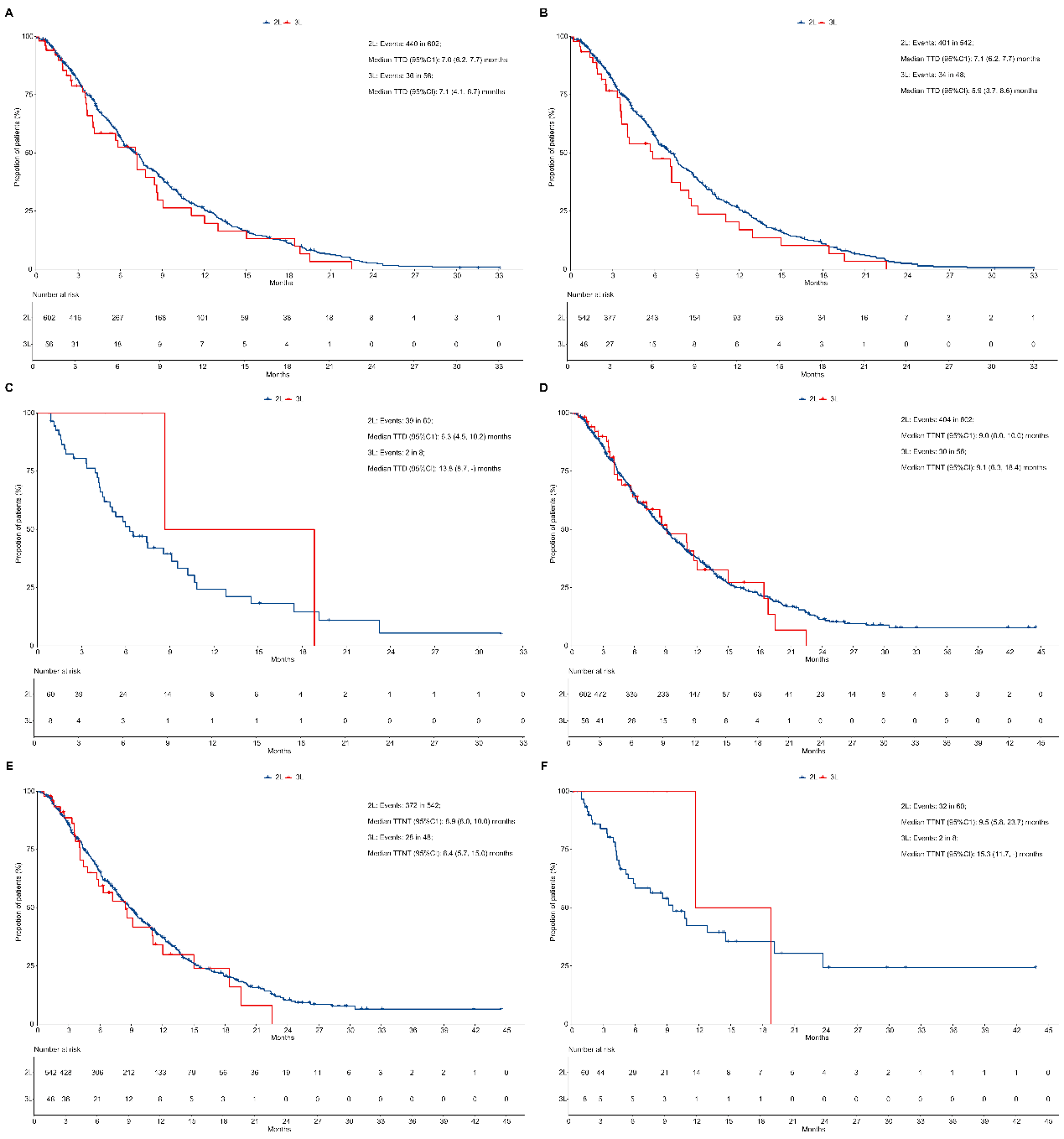

Supplement: Supplementary file 1 — Supplementary Material 1 [file 10585_2026_10417_MOESM1_ESM.docx]
